# Editorial: Unveiling the role of carbohydrates in cardiometabolic health

**DOI:** 10.3389/fnut.2025.1568319

**Published:** 2025-02-20

**Authors:** Debora Collotta, Gustavo Ferreira Alves

**Affiliations:** ^1^Department of Clinical and Biological Sciences, University of Turin, Turin, Italy; ^2^Pharmacology Unit, School of Pharmacy, University of Camerino, Camerino, Italy

**Keywords:** carbohydrates, cardiometabolic health, diet, AGEs, fructose

The intricate relationship between dietary carbohydrates and cardiometabolic health has long been a focal point of nutritional science and medical research. As the cornerstone of the global diet, carbohydrates have a profound influence on energy metabolism, glycemic control, and cardiovascular outcomes. However, not all carbohydrates are created equal and understanding their diverse roles is pivotal in addressing the rising prevalence of cardiometabolic disorders such as diabetes, obesity, and cardiovascular disease. In this context, and building upon our previous contributions to the field, we have employed various approaches to explore metabolic disorders. Our research has included presenting evidence on the detrimental effects of advanced glycation end-products (AGEs) in the development of metabolic conditions (PMID: 32824970), as well as investigating potential pharmacological strategies for mitigating diet-related metaflammation (PMID: 32413585). Moreover, our studies have demonstrated the protective effects of a nutritional supplementation regimen using the microalga *Arthrospira platensis* (spirulina, SP), showing its potential in reducing metabolic inflammation (PMID: 38398877).

Given our expertise in these areas, we firmly believe that this Research Topic is both timely and necessary. This Research Topic, “*Unveiling the role of carbohydrates in cardiometabolic health*,” aimed to shed light on the complex interplay between carbohydrate consumption and cardiometabolic health outcomes, presenting a collection of cutting-edge research and reviews that explore this crucial subject from multiple perspectives. Recent decades have seen an explosion of interest in the quality and quantity of carbohydrate intake. While refined carbohydrates and added sugars have been implicated in adverse health outcomes, whole-grain carbohydrates, dietary fiber and resistant starches are increasingly recognized for their protective effects. The articles featured in this Research Topic investigate these nuances, offering evidence-based insights into how carbohydrate quality and source impact metabolic pathways and disease risk. Collectively, these contributions challenge simplistic categorizations of carbohydrates and emphasize the importance of context, including individual metabolic responses, dietary patterns, and cultural differences, in determining their health effects.

One of the fundamental articles in this Research Topic, Pointke et al., investigates the acute effects of isocaloric diets with different macronutrient compositions (high-fat, high-carbohydrate, and control) on glucose metabolism and hedonic regulation. The study finds that a short-term high-fat diet leads to lower postprandial glucose and insulin levels compared to the high-carbohydrate and control diets. Moreover, the high-fat diet reduced insulin resistance.

On the other hand, no significant changes were observed in hedonic appetite regulation across diets. These findings provide relevant insights into how macronutrient composition in short-term diets can influence glucose metabolism without affecting hedonic food intake regulation. This underscores the importance of dietary balance in maintaining metabolic health and suggests that high-fat diets may have therapeutic potential for the short-term management of glucose tolerance impairments.

Building on the exploration of carbohydrate impacts, Semchyshyn investigates the role of fructose in promoting advanced glycation end-products (AGEs) and their interaction with receptors for AGEs (RAGE). With the knowledge that excessive long-term fructose consumption can have detrimental health effects, this study reviewed how AGEs, which can be produced by fructose-initiated non-enzymatic glycation (fructation), contributed to upregulation of their own receptor (RAGE) and amplify RAGE-mediated signaling related to inflammation, metabolic disorders, chronic diseases, and aging. It highlights that downregulation of the AGE-RAGE axis through lifestyle improvements might be a promising non-pharmacological strategy for decreasing disease conditions associated with RAGE-mediated inflammation, thus suggesting the potential for mild modulation approaches to manage cardiometabolic risks. A historical perspective is provided by Ting, which revisits Yudkin's groundbreaking hypothesis, first proposed in the 1970s, that dietary sugar, particularly fructose, is a major contributor to cardiovascular disease (CVD). It highlights how contemporary research supports this claim through an examination of fructose metabolism and its metabolic consequences. Key findings in this interesting study include a cross-talk between fructose and aterosclerosis, inflammatory mechanisms, insulin resistance and lipogenesis, epidemiological evidence and reevaluation of dietary guidelines. Yudkin's hypothesis is increasingly supported by modern evidence, with fructose overconsumption emerging as a significant factor in CVD progression. The review calls for further research on fructose's metabolic impacts and its inclusion in public health strategies to reduce cardiovascular risk.

Further expanding our understanding between diet and sleep quality, the article by Cao et al. explores through a cross-sectional study the relationship between Relative Fat Mass (RFM), Low-Carbohydrate Diet (LCD) scores, and sleep disorders using data from the National Health and Nutrition Examination Survey (NHANES). The main finding highlights the impact of body composition and carbohydrate intake on sleep disorders. For instance, both RFM and LCD scores were positively associated with the risk of sleep disorders. Participants with higher RFM or LCD scores showed significantly greater odds of experiencing sleep disturbances. The authors demonstrated significant public health implications, and that high RFM and LCD scores may serve as independent risk factors for sleep disorders. This highlights the importance of addressing obesity and dietary patterns in interventions for improving sleep health. This study provides evidence that obesity and dietary habits, particularly adherence to low-carbohydrate diets, are significant factors in the development of sleep disorders. It suggests promoting balanced diets and weight management as strategies to mitigate these risks.

Finally, Volek et al. synthesize expert perspectives to provide practical dietary recommendations. The consensus suggests to the Dietary Guidelines for Americans (DGA) more flexibility by incorporating a broader range of carbohydrate intake options that support overall health. Specifically, describing that it could introduce a lower-carbohydrate eating pattern that is culturally relevant and fosters health equity across diverse populations, alongside the current healthy dietary patterns. Furthermore, supported by research data, the consensus suggests that a well-designed low-carbohydrate diet could help reduce the high rates of obesity, prediabetes, metabolic syndrome, and Type 2 diabetes, while also advancing food security and health equity, making it a valuable addition to the DGA recommendations. The consensus, thus emphasizes the need for equity in dietary guidance, advocating for approaches that consider socioeconomic and cultural factors alongside evidence-based insights.

A key objective of this Research Topic is to bridge the gap between fundamental research and practical application. By integrating findings from these studies, clinicians, and researchers can develop more nuanced dietary recommendations that consider individual variability, cultural practices, and broader public health implications.

Placing this work in a broader context, it is clear that carbohydrates are at the center of several global health challenges. The double burden of malnutrition, which involves the coexistence of undernutrition and overnutrition, underscores the urgent need for more nuanced discussions on carbohydrate consumption. Policymakers and healthcare providers must navigate conflicting dietary paradigms and cultural traditions to create interventions that are both effective and culturally sensitive. The findings presented here provide valuable insights for such efforts, equipping stakeholders with the evidence needed to make informed decisions.

The editors extend their gratitude to the authors and reviewers whose contributions have made this Research Topic possible. By exploring the multifaceted roles of carbohydrates in cardiometabolic health, this Research Topic, not only advances scientific understanding but also promotes a more holistic approach to dietary recommendations and public health strategies. We hope that this editorial and the accompanying articles will inspire further research and dialogue in this important area of study.

